# Tetrachloroethylene (PCE, Perc) Levels in Residential Dry Cleaner Buildings in Diverse Communities in New York City

**DOI:** 10.1289/ehp.7414

**Published:** 2005-06-21

**Authors:** Michael J. McDermott, Kimberly A. Mazor, Stephen J. Shost, Rajinder S. Narang, Kenneth M. Aldous, Jan E. Storm

**Affiliations:** 1 Center for Environmental Health, New York State Department of Health, Troy, New York, USA; 2 Wadsworth Center for Laboratories and Research, New York State Department of Health, Albany, New York, USA

**Keywords:** dry cleaners, environmental justice, PCE, perc, race/ethnicity, socioeconomic status, tetrachloroethylene

## Abstract

Fugitive tetrachloroethylene (PCE, perc) emissions from dry cleaners operating in apartment buildings can contaminate residential indoor air. In 1997, New York State and New York City adopted regulations to reduce and contain perc emissions from dry cleaners located in residential and other buildings. As part of a New York State Department of Health (NYSDOH) study, indoor air perc levels were determined in 65 apartments located in 24 buildings in New York City where dry cleaners used perc on site. Sampling occurred during 2001–2003, and sampled buildings were dispersed across minority and nonminority as well as low-income and higher income neighborhoods. For the entire study area, the mean apartment perc level was 34 μg/m^3^, 10-fold lower than mean apartment levels of 340–360 μg/m^3^ documented before 1997. The maximum detected perc level was 5,000 μg/m^3^, 5-fold lower than the maximum of 25,000 μg/m^3^ documented before 1997. Despite these accomplishments, perc levels in 17 sampled apartments still exceeded the NYSDOH residential air guideline of 100 μg/m^3^, and perc levels in 4 sampled apartments exceeded 1,000 μg/m^3^. Moreover, mean indoor air perc levels in minority neighborhoods (75 μg/m^3^) were four times higher than in nonminority households (19 μg/m^3^) and were > 10 times higher in low-income neighborhoods (256 μg/m^3^) than in higher income neighborhoods (23 μg/m^3^). Logistic regression suitable for clustered data (apartments within buildings) indicated that perc levels on floors 1–4 were significantly more likely to exceed 100 μg/m^3^ in buildings located in minority neighborhoods (odds ratio = 6.7; 95% confidence interval, 1.5–30.5) than in nonminority neighborhoods. Factors that may be contributing to the elevated perc levels detected, especially in minority and low-income neighborhoods, are being explored.

Tetrachloroethylene (PCE), commonly referred to as perc, is the most frequently used solvent in the dry cleaning industry (Earnest 1996). In New York City and many other urban areas, dry cleaners using perc are sometimes colocated with residences, offices, retail businesses, or food establishments and emit fugitive perc emissions that contaminate indoor air throughout the buildings where they are located (Schreiber et al. 1993, 2002; Wallace et al. 1995). Perc levels in buildings with an operating dry cleaner, or simply near a dry cleaner, have ranged up to 55,000 μg/m^3^ (Altmann et al. 1995; Schreiber et al. 1993, 2002; Wallace et al. 1995).

In the workplace, air perc levels averaging about 30,000–80,000 μg/m^3^ have been associated with alterations in color vision and cognitive function (Gobba 2000), and levels of 1,800–2,400 μg/m^3^ have been reported to decrease visual contrast sensitivity (VCS) (Schreiber et al. 2002). Residential indoor air perc levels averaging about 5,000 μg/m^3^ have been associated with small but statistically significant deficits in cognitive performance (e.g., deficits in short-term memory, decreased reaction time) (Altmann et al. 1995), and residential indoor air levels averaging about 700 μg/m^3^ have been associated with decreases in visual function, although decreases were not significant, and residents’ function was still within a normal range (Schreiber et al. 2002; Storm and Mazor 2004).

These observations together have raised concern that residents of buildings where dry cleaners are using perc on site (i.e., residential dry cleaner buildings) may experience long-term, involuntary, and possibly harmful perc exposures. Based on this concern and evaluation of visual and other health effects associated with perc exposure, the New York State Department of Health (NYSDOH) derived a health-based guideline of 100 μg/m^3^ perc for residential air, considering continuous lifetime exposure and sensitive people (NYSDOH 1997, 2003). The NYSDOH currently considers this level to be a useful guideline in aiding decisions about the nature and urgency of efforts to reduce residential exposures to perc. Actions to reduce exposure are recommended by the NYSDOH if perc levels are above background even if they are < 100 μg/m^3^, but an increase in the scale and urgency of such actions is recommended when air levels are > 100 μg/m^3^. The NYSDOH recommends immediate action when an air level is ≥ 1,000 μg/m^3^.

Perc exposures have also been addressed by the federal government. In 1993, the U.S. Environmental Protection Agency issued regulations to control air emissions of perc from dry cleaners (U.S. Environmental Protection Agency 1993). However, these regulations did not specifically address fugitive perc emissions from dry cleaners in residential buildings. Hence, the New York State Department of Environmental Conservation (NYSDEC) and the New York City Department of Environmental Protection (NYCDEP) adopted additional dry cleaner regulations intended to reduce and contain fugitive perc emissions in 1997 and 1998, respectively, which specifically addressed dry cleaners in residential buildings (New York City 1998; NYSDEC 1997). Deadlines for compliance with specific components of the regulations were staggered over several years depending upon the type (“generation”) of dry cleaning equipment being used and the type of building (commercial or mixed use) where the dry cleaner was located. The dry cleaner regulations also mandated training and required submission of annual inspection reports by state-approved, third-party inspectors that are used to help document compliance.

Concurrent with adoption of these additional dry cleaner regulations, the NYCDEP and the New York City Department of Health and Mental Hygiene (NYCDOHMH) initiated a process to specifically address complaints from apartment building residents concerned about perc emissions from dry cleaners. Upon receipt of a citizen complaint regarding perc, the NYCDOHMH determines indoor air perc levels in complainants’ residences. Depending upon the level of perc detected, the dry cleaning equipment is sealed (perc > 1,000 μg/m^3^) or a notice of violation to the dry cleaner operator is issued (100 μg/m^3^ < perc < 1,000 μg/m^3^). In either case, the NYCDEP conducts an on-site investigation of the dry cleaner to determine compliance with dry cleaner regulations and to identify remedial actions required to reduce fugitive perc emissions. This complaint response process is a valuable component of dry cleaner regulation enforcement in New York City while also providing anecdotal information on perc levels in “complaint” buildings.

In 2000, the NYSDOH began recruitment for the New York City Perc Project (NYC Perc Project), a study intended to document perc exposures and possible associated visual function effects among residents of dry cleaner buildings. Indoor air perc levels and biologic (exhaled breath, blood) measures of perc exposure were obtained for residents in buildings with and without dry cleaners, and visual function was assessed using measures of VCS and color vision, previously shown to be adversely affected by perc or solvent exposure (Frenette et al. 1991; Gobba 2000; Iregren et al. 2002; Mergler 1991; Mergler and Blain 1987; Mergler et al. 1987, 1996; Schreiber et al. 2002).

Indoor air sample collection and analyses for the NYC Perc Project began in 2001, coincidentally midway through full implementation of the state and city dry cleaner regulations adopted in 1997–1998. The earliest sampled dry cleaner buildings had indoor air perc levels that were markedly below levels reported before 1997 (Schreiber et al. 2002; Wallace et al. 1995), with the unexpected exception of buildings located in neighborhoods with large minority and/or low-income populations. Although the NYC Perc Project was not specifically designed to evaluate the influence of neighborhood socioeconomic characteristics or state and city dry cleaner regulations on indoor air perc level in residential dry cleaner buildings, the results of this sampling effort provide a valuable initial basis for doing this and are reported here. The findings described should prove helpful in continuing federal, state, and local efforts to ensure that residential perc exposures are appropriately limited for all those residing in buildings with dry cleaners using perc.

## Materials and Methods

### Study area and building selection.

Eleven ZIP code areas surrounding Central Park in the borough of Manhattan in New York City comprised the main study area. These areas were selected largely based on their high density of residential dry cleaner buildings, the presence of some buildings where residential perc levels up to 5,000 μg/m^3^ had been previously documented (NYSDOH, unpublished data; Schreiber et al. 2002), and their close proximity to the location of participant visual function evaluations at the Mount Sinai School of Medicine. Coincidentally, these ZIP code areas also encompass neighborhoods characterized by markedly different income and minority characteristics.

Most dry cleaners in residential buildings included in this study were identified from registration certificates submitted to the NYSDEC as required by the 1997 dry cleaner regulations. Some others were identified from NYSDEC National Emissions Standards for Hazardous Air Pollutants (NESHAP) for Perchloroethylene Dry Cleaners records and from Internet-based business directories (ReferenceUSA, InfoUSA Inc., Omaha, NE; InfoSpace, InfoSpace Inc., Bellevue, WA). Internet-based business directories were cross-referenced against NYSDEC records to ensure that all dry cleaners in the study area were identified, because not all dry cleaners complied with NESHAP or NYSDEC reporting requirements. Dry cleaners identified were contacted by telephone to ascertain whether they were still in business and whether they identified themselves as using perc on site or as drop-off facilities (i.e., locations where items to be dry cleaned are dropped off and picked up but no dry cleaning occurs on site).

Identified dry cleaner buildings were visited and characterized from the sidewalk to verify that the dry cleaner was operating on site and that occupied residences were present in the same building. Numbers of residential floors were also noted for each building. Because NYC Perc Project inclusion criteria required that participants have no exposure to volatile organic compounds (VOCs) other than perc that might influence visual function, dry cleaner buildings where other businesses using VOCs (e.g., nail salons, shoe repair stores, photography developing) were present were excluded from further consideration. At least three other residential buildings with no dry cleaner or other business possibly using VOCs, and located at least one city block away from each dry cleaner building meeting inclusion criteria, were identified as reference buildings.

Early analytical results indicated that indoor air perc levels in most apartments in dry cleaner buildings sampled were below, or only slightly above, the NYSDOH residential air guideline of 100 μg/m^3^. Higher levels were found in dry cleaner buildings located in low-income, minority neighborhoods and in buildings elsewhere that had been the subject of a resident complaint. Because successful completion of the NYC Perc Project required that as many apartments as possible with elevated perc levels be identified, the strategy for identifying buildings for inclusion was modified so that buildings located in minority or low-income ZIP code areas and those that had been the subject of a complaint were prioritized.

Several residential buildings with dry cleaning drop-off facilities were inadvertently included early in the study before phone calls to ascertain whether dry cleaners were using perc on site were instituted. Although not meeting study criteria for inclusion in the NYC Perc Project, indoor air perc levels associated with drop-off facilities are of interest and so are also reported here.

### Household recruitment and participant activities.

Buildings sampled include residential buildings where at least one household met NYC Perc Project eligibility criteria and enrolled in the study. Eligible households included those with one adult (20–55 years of age) and at least one child (5–14 years of age) residing in their building for at least 1 year. Adult–child pairs meeting these criteria and willing to participate were initially screened to exclude those with current or previous exposures to VOCs and/or medical conditions that could possibly interfere with visual function evaluation (i.e., diabetes, cataracts, glaucoma). Indoor air in five households without children was also sampled because residents were concerned and adamant about having their indoor air tested or because residents participated early in the study to help optimize study procedures. During screening, participants were asked to categorize their household race/ethnicity into one or more (up to four) of the following categories: white, African American, American Indian, Chinese, Japanese, Korean, Native Hawaiian, Samoan, Hispanic, or other. Adult participants were also asked to categorize their annual household income into one of the following ranges: < $15,000, $15,000–30,000, $30,000–45,000, $45,000–60,000, or > $60,000.

In most ZIP code areas, written material describing the NYC Perc Project was mailed to apartments in targeted residential dry cleaner buildings using addresses obtained during visits to the building or through the New York State Zip+4 Directory (U.S. Postal Service 2000). Listed telephone numbers associated with targeted buildings were obtained through reverse address queries from Internet-based residential telephone directories (ReferenceUSA, InfoSpace). Up to five calls to every residential telephone number were made at different times of day and on different days of the week beginning 5 days after study information had been mailed to addressees. Messages describing the study were left on all answering machines encountered. When a telephone call was answered, an attempt was made to determine whether an adult–child pair was present. If so, the respondent was asked to complete the screening questionnaire.

In ZIP code areas that have large minority (either predominately Hispanic or predominately African American) populations, recruitment was conducted through door-to-door visits by bilingual (Spanish/English) community health workers. This approach is consistent with recommendations for recruiting minority and lower-income populations (Cabral et al. 2003; Fitzgibbon et al. 1998; Grunbaum et al. 1996; Harris et al. 2003). Community health workers visited all residences in targeted buildings during afternoon and evening hours on different days of the week. Adults responding to door knocks were given a verbal description of the study and a written fact sheet describing the project, in Spanish or English, whichever was appropriate, and were administered the screening questionnaire. Written information urging residents to call the NYC Perc Project to enroll or obtain more information was left on doorsteps or slipped under doors when residents were not at home.

Residences of all eligible participants were visited to collect 24-hr indoor air samples. During these home visits, other activities associated with the NYC Perc Project also occurred (e.g., collection of exhaled breath samples, completion of residential/occupational/medical history questionnaires). All participants volunteered and signed adult consent and/or child assent forms approved by the NYSDOH and the Mount Sinai School of Medicine institutional review boards. Participants received $100 to compensate them for their participation in the NYC Perc Project, screening for glaucoma and other eye diseases, and a prescription for corrective lenses, if warranted, at no cost.

### Indoor air sample collection and analysis.

Indoor air samples were collected using 3M organic vapor monitors (3M, St. Paul, MN) deployed in duplicate in the main living areas. Monitors were placed approximately 6 feet high and away from any direct sources of ventilation such as windows, air conditioners, fans, or heating/cooling vents. Air sampling occurred for 21–27 hr during weekdays beginning between 1500 and 2100 hr. A hard plastic, impermeable lid provided by the manufacturer was affixed to each monitor at the end of the collection period.

Monitors were analyzed for perc by the NYSDOH Wadsworth Center for Laboratories and Research in Albany, New York, as described by Amin et al. (1998). Analytical results were reviewed at the laboratory in accordance with approved quality assurance/quality control procedures and entered into the NYS-DOH Environmental Laboratory Data Accessioning and Reporting System. Sample results at or below the detection limit of 5 μg/m^3^ are reported as present but less than 5 μg/m^3^ (PL). Both the participating household and the NYCDOHMH were notified as soon as possible when apartment perc levels were above background, and follow-up activities were initiated by the NYCDOHMH.

### Geographic information system application.

Buildings were geocoded according to street address using MapInfo (professional version 7.0; MapInfo Corporation, Troy, NY) and were assigned Census 2000 (U.S. Census Bureau 2002) block group characteristics for the census block group where they were located. Census block groups were categorized as minority or low income according to criteria for New York State urban areas and New York State urban poverty thresholds, respectively, outlined in the NYSDEC Environmental Justice and Permitting Policy (NYSDEC 2003). Census block groups with a population ≥51.1% Hispanic, African American, Asian and Pacific Islander, or American Indian (or < 51.1% non-Hispanic white) were classified as minority. Census block groups in which ≥23.59% of the population fell below the poverty threshold were classified as low income.

### Analyses.

Quantities of perc in indoor air present but below the detection limit of 5 μg/m^3^ were assigned half the detection limit, and duplicate samples were averaged to determine apartment perc level. We evaluated apartment perc levels qualitatively against background levels of perc and against the NYSDOH residential air guideline of 100 μg/m^3^. Background was considered to be ≤11 μg/m^3^, the 75th percentile of indoor air perc levels detected in homes and offices sampled throughout the United States (Shah and Heyerdahl 1988). We also qualitatively compared perc levels with those measured in residential dry cleaner buildings before 1997 before adoption of state and city dry cleaner regulations.

We used Pearson’s correlation coefficients to estimate the association between resident self-reported race/ethnicity [minority (i.e., non-Hispanic white), nonminority (i.e., not non-Hispanic white)] and annual income range (< $30,000/year, > $30,000/year), and census block group assignment of residents’ building. We used logistic regression using generalized estimating equations appropriate for clustered observations, and SAS software (release 9.1; SAS Institute, Cary, NC) to evaluate associations between the occurrence of indoor air perc levels greater than the NYS-DOH residential air guideline of 100 μg/m^3^ and building census block group income or minority category.

## Results

Both building and household inclusion criteria influenced which buildings and apartments were sampled as illustrated in Table 1. Overall, 180 dry cleaner facilities reported using perc on site. Of these, 136 were characterized to determine whether they met building inclusion criteria. Eighty-three met inclusion criteria, recruitment of households was attempted in 67, and sampling occurred in at least one apartment in 24. Although there were comparatively fewer dry cleaner buildings present in minority, low-income ZIP code areas, they accounted for a third of all dry cleaner buildings sampled. This reflects the comparatively larger proportion of households in these buildings meeting household inclusion criteria, as also noted in Table 1 and discussed further below. Also, nine sampled buildings had been the subject of a prior complaint, all of which were located in nonminority, higher income ZIP code areas. At least one household in 36 residential buildings without a dry cleaner was also sampled.

The study requirement that sampled households include an adult and child clearly influenced the sample obtained. As illustrated in Table 1, only about 1 in 10 households contacted included an age-eligible child (i.e., were potentially eligible). A higher proportion of contacted households in minority, low-income ZIP code areas had age-eligible children, so this study requirement contributed to a higher proportion of potentially eligible households being identified in minority, low-income ZIP code areas. This, combined with comparatively higher eligibility and participation rates, contributed to the final sample in which one-third of sampled households in dry cleaner buildings were in minority, low-income ZIP code areas even though they accounted for only about 1/10th of total contacted households. Recruitment and enrollment of contacted households in buildings without dry cleaners showed similar patterns. Also, 21 sampled households in nonminority, higher income ZIP code areas were in buildings that had been the subject of a prior complaint.

To assess the potential for selection bias given the low household contact and eligibility rates illustrated in Table 1, every dry cleaner building in the study area was assigned socioeconomic and demographic characteristics of the census block group where it was located. Population characteristics associated with dry cleaner buildings that were sampled and those that were not sampled were similar. In nonminority, higher income ZIP code areas, sampled dry cleaner buildings were located in census block groups averaging 74% nonminority and 7% low-income populations, and in which 7% of households included children 5–15 years of age. Dry cleaner buildings characterized, meeting inclusion criteria, and subjected to recruitment that were not sampled were in census block groups averaging 78% nonminority and 8% low-income populations and in which 7% of households included children 5–15 years of age. In minority, low-income ZIP code areas, sampled dry cleaner buildings were located in census block groups averaging 25% nonminority and 21% low-income populations and in which 14% of households included children 5 and 15 years of age. Dry cleaner buildings characterized, meeting inclusion criteria, and subjected to recruitment that were not sampled were in census block groups averaging 43% nonminority and 15% low-income populations and in which 11% of households included children 5–15 years of age. In all ZIP code areas, census block group characteristics assigned to dry cleaner buildings that were not characterized, that did not meet inclusion criteria, and/or that were not subjected to recruitment were similar to characteristics assigned to buildings that were sampled. Thus, within ZIP code areas, population characteristics of the dry cleaner buildings sampled are similar to those that were not sampled. Additionally, building census block group assignment and self-reported household minority and income categories were significantly correlated for building and household minority category (r = 0.55, p < 0.0001) and for building and household low-income category (r = 0.48, p = 0.005). Thus, socioeconomic characteristics of building census block group assignment and building residents appear to be equivalent, and characteristics associated with sampled buildings appear to be similar to other dry cleaner buildings in the same ZIP code areas.

Table 2 details minority and income census block group assignment for each dry cleaner building sampled as well as whether it had ever been the subject of a complaint, number of floors in each building, and perc levels for each household sampled. Table 2 conveys the following information pertinent to interpreting indoor air perc levels in the dry cleaner buildings sampled. First, the buildings sampled are dispersed throughout minority, low-income and nonminority, higher income neighborhoods and thus provide information for buildings in socioeconomically diverse areas. Second, the six highest perc levels detected, ranging between 695 and 5,000 μg/m3, are in six different dry cleaner buildings located in census block groups characterized as minority or as both minority and low income. These buildings are also among the smallest buildings sampled, only one having more than four floors (Table 2). Third, perc levels in “complaint” buildings, ranging from 5 (PL) to 372 μg/m3, were not among the highest in the study area, although they were among the highest in nonminority, higher income census block groups. None of the nine “complaint” buildings sampled was in a minority or low-income area. Fourth, all residences with perc > 100 μg/m3, with one exception (building e47), occurred on floors 1–4 of sampled buildings (Table 2). Finally, 12 of the 24 sampled dry cleaner buildings had at least one apartment where perc levels were > 100 μg/m3, with four of them also having at least one apartment where perc levels were > 1,000 μg/m3 (Table 3).

Importantly, when only buildings located in minority and/or low-income neighborhoods are considered, mean (geometric) perc levels are close to or exceed the NYSDOH residential air guideline of 100 μg/m3. Table 4 shows that indoor air perc level in 29 apartments in 10 dry cleaner buildings located in a minority census block group averaged 76 μg/m3, compared with 19 μg/m3 in 36 apartments in 14 buildings located in nonminority census block groups. The mean perc level in 11 apartments in 5 dry cleaner buildings located in a low-income census block group was 256 μg/m3, compared with 23 μg/m3 in 54 apartments in 19 buildings located in non-low-income census block groups. Thus, residents of dry cleaner buildings in minority, low-income areas appear to have disproportionately elevated exposures to perc even though, overall, perc levels have decreased since adoption of the 1997 dry cleaner regulations.

## Discussion

We determined indoor air perc levels in residential buildings with on-site dry cleaners and in nearby residential buildings without dry cleaners in the borough of Manhattan, New York City. Buildings sampled included only those that were evaluated for NYC Perc Project inclusion and that met building inclusion criteria (e.g., no other source of VOCs present, occupied residences present). Additionally, individual apartments sampled included mostly those meeting NYC Perc Project household inclusion criteria (i.e., presence of an adult and child residing in the same household for at least 1 year with no other VOC exposures or certain medical conditions), although five sampled apartments included only adult residents. Thus, the sample obtained is not a truly random sample of all dry cleaner buildings in the study area. However, socioeconomic characteristics of the census block groups where sampled buildings are located reflect socioeconomic characteristics of their larger ZIP code area, are equivalent to census block groups where buildings that were not sampled are located, and are correlated with sampled household self-reported socioeconomic characteristics. Thus, conclusions drawn with respect to sampled building neighborhood characteristics and indoor air perc level are likely to be applicable to other residential buildings matching NYC Perc Project building inclusion criteria (e.g., dry cleaner using perc on site; no other source of VOCs).

Results demonstrate that mean indoor air perc levels in residential dry cleaner buildings in the study area have decreased by about 10-fold overall since adoption of state and city dry cleaner regulations (Table 3) and related enforcement activities (e.g., the complaint response process) in 1997. Maximum indoor air perc values have decreased about 5-fold over the same period. The range of perc levels observed in this study is also lower than the range of levels recently found in a jurisdiction without additional, nonfederal dry cleaner regulations in place. In eight residences in dry cleaner buildings in Hudson County, New Jersey, selected randomly from a list of 82 dry cleaners colocated with residences, indoor air perc levels ranged from 470 to 4,200 μg/m3 when sampled in 1998 (Garetano and Gochfeld 2000). By comparison, perc levels in most residences in dry cleaner buildings reported here were < 400 μg/m3, although eight apartments had perc levels > 400 μg/m3 (Table 2).

Thus, the findings reported here indicate that state and city dry cleaner regulations that specifically address the control of fugitive perc emissions from dry cleaners operating in residential buildings have apparently contributed to a substantial decrease in indoor air perc levels in those buildings. It is not clear how large a role, if any, the complaint response process has played in this decrease. Data were not obtained in this study that would support analysis of this. Moreover, despite the overall decrease in perc levels, mean levels in dry cleaner buildings remain elevated above levels in buildings with only drop-off facilities or without a dry cleaner (Table 4). Additionally, half the residential dry cleaner buildings sampled still had at least one apartment where indoor air perc levels exceeded the NYSDOH residential air guideline of 100 μg/m3, and four of them had at least one apartment where perc levels exceeded 10 times the NYSDOH residential air guideline (Tables 2 and 3). Of the 65 individual apartments sampled, 17 had perc levels > 100 μg/m3, and 4 had a perc level > 1,000 μg/m3 (Table 3). Thus, despite the evident success of additional dry cleaner regulations adopted in 1997 in reducing residential exposures to perc, involuntary residential perc exposures continued in the study area, at least through 2003, when sampling for this study was completed.

Importantly, the decrease in perc levels occurred unevenly. Perc levels were disproportionately higher in residential dry cleaner buildings located in minority, low-income neighborhoods compared with nonminority, higher income neighborhoods (Tables 2 and 4). All 4 apartments with perc levels > 1,000 μg/m3 are located in 4 different dry cleaner buildings in minority neighborhoods (3 of which are also low income), whereas none of 36 apartments in 14 dry cleaner buildings in nonminority, higher income neighborhoods had perc levels > 1,000 μg/m3 (Table 2). Further, mean perc levels in dry cleaner buildings in low-income or minority neighborhoods are about 10 and 4 times higher than mean levels in higher income and nonminority neighborhoods, respectively (Table 4). Finally, logistic regression indicated a significantly increased likelihood that apartments on lower floors in residential dry cleaner buildings located in minority neighborhoods would have perc levels > 100 μg/m3 compared with apartments in residential dry cleaner buildings located in nonminority neighborhoods. Individual household race/ethnicity and annual income were significantly correlated with residents’ building census block group minority and income assignment, providing corroborative evidence that minority, low-income residents of dry cleaner buildings have disproportionately elevated exposures to perc compared with nonminority, higher income residents.

Such disproportionate exposures of minority, low-income subpopulations is consistent with other recent reports that minority and low-income communities experience greater exposures to hazardous environmental contaminants than do other communities (Bowen 2002; Maantay 2002). However, most such reports of inequities in environmental exposures rely on estimates of exposure to hazardous substances based on geographic proximity of minority and low-income neighborhoods to potential sources of hazardous substances (e.g., Superfund sites, industrial facilities, etc.). Here, spatial analyses of small-area contaminant sources (e.g., dry cleaners) were combined with information about neighborhood minority and income characteristics (e.g., census block group data) and individual exposure estimates (e.g., apartment perc level) to document that, indeed, individual minority, low-income residents of dry cleaner buildings are likely to have greater perc exposure than are other residents of dry cleaner buildings.

It is not known why indoor air perc levels exceeded 100 μg/m3, and even 1,000 μg/m3, in some residential dry cleaner buildings 6 years after adoption of regulations intended to control them. One possible contributing factor is inconsistent or poor compliance with dry cleaner regulations by dry cleaners in affected buildings. Information provided to the NYSDEC by dry cleaners in the buildings sampled, as required by the dry cleaner regulations, indicates that dry cleaners in 22 of the 24 sampled buildings were using equipment that was in compliance with the regulations at the time of sampling (information was unavailable for dry cleaners in two sampled buildings, e368 and e6). Thus, it does not appear that a failure to use approved dry cleaner equipment accounted for these observations. Work practices (e.g., maintenance of effective vapor barrier/room enclosure, proper use of exhaust fans) can influence fugitive perc emissions, and prior complaints associated with some of the dry cleaner buildings sampled, even though equipment met regulatory requirements, suggests that poor work practices may have contributed to some of the elevated perc levels observed. This is consistent with another recent report that elevated perc levels (420–7,200 μg/m3) occurred in residences colocated with dry cleaners even though dry cleaning equipment met or exceeded federal dry cleaner regulatory requirements, and that positive work practices were associated with comparatively lower perc levels (Garetano and Gochfeld 2000). Regulatory agencies involved in dry cleaner regulation enforcement in New York (NYSDEC, NYSDOH, NYCDEP, NYCDOHMH) are currently evaluating these possibilities.

Another possible contributing factor to the higher perc levels found in some residential dry cleaner buildings is the existence of undesirable air flow and ventilation characteristics, especially in older buildings. Indoor air quality investigations in residences colocated with dry cleaners completed by state and city staff frequently note higher perc levels where there are structural conditions providing pathways for perc migration (e.g., poorly sealed pipe chases, cracks in walls or ceilings). Associations between substandard housing and increased exposure to environmental tobacco smoke, lead, mold, and pesticides is well recognized (Breysse et al. 2004; Krieger and Higgins 2002), but associations between substandard residential building quality and levels of indoor air contaminants originating from a source outside the home, such as a nearby dry cleaner, have not yet been thoroughly investigated. The findings here should encourage such an examination.

Finally, residents of buildings in minority, low-income neighborhoods may be less likely to complain to the city about fugitive perc emissions from a dry cleaner in their building. As noted above, the complaint response process is a valuable tool the city health department uses to help identify instances where residential perc levels are elevated and consequently where dry cleaners may not be operating in compliance with regulations. The observation that none of the sampled dry cleaner buildings in minority, low-income areas had ever been the subject of a prior complaint whereas 9 of the 16 sampled dry cleaner buildings in the remainder of the study had been, is consistent with this notion. On the other hand, some of the “complaint” buildings had some of the highest perc levels in nonminority, higher income areas. Thus, it is not clear whether the complaint response process contributed to reductions of perc to ≤100 μg/m3. Unfortunately, additional data were not gathered during this study that would allow an evaluation of the relationship between resident complaints and residential perc levels.

Bias in the selection of households sampled could have influenced the results in the observed direction if recruitment methods reduced the likelihood of including apartments with elevated perc levels in nonminority, higher income neighborhoods. However, it appears unlikely that this occurred to a major extent. Although not all residential dry cleaner buildings were targeted for recruitment in nonminority, higher income neighborhoods, many of those that were targeted were “complaint” buildings and were therefore thought most likely to have elevated perc levels. Nine of the 17 buildings sampled in these areas had been the subject of a prior complaint, and indeed, they were among the 4 buildings in these areas with the highest perc levels (Table 2). Bias may also have influenced results in the observed direction if recruitment methods increased the likelihood of including apartments with elevated perc in minority, low-income neighborhoods. This also appears unlikely to have significantly influenced results. Although a higher proportion of apartments on floors 1–4 in minority and/or low-income neighborhoods were sampled compared with nonminority, higher income neighborhoods, similar numbers of samples on floors 1–4 were obtained in both areas and the highest absolute levels of perc were consistently observed in minority, low-income areas (Table 2). Further, participation rates were similar for eligible households in both socioeconomic neighborhoods, providing no suggestion that those with comparatively higher or lower levels of perc were more or less likely to participate (Table 1). Still, the possibility that differences in recruitment strategies or other characteristics differentiating minority, low-income households from nonminority, higher income households may have influenced these findings is an acknowledged limitation of this study.

It is not known whether adverse health effects are associated with the levels of residential indoor air perc reported here, or whether adverse health effects may be associated with them in the future. In one study, 14 adults living in apartments near dry cleaning shops had significantly reduced scores on tests of cognitive function compared with age- and sex-matched controls (Altmann et al. 1995). The range of indoor perc level was 8–23,000 μg/m3, the median (and geometric mean) was 1,400 μg/m3, and the arithmetic mean was 5,000 μg/m3. Another study of 13 adult residents of dry cleaner buildings found that VCS and color discrimination ability were decreased, although they did not differ significantly from that of age- and sex-matched controls (Schreiber et al. 2002; Storm and Mazor 2004). Perc levels in apartments of tested adults averaged 1,800 μg/m^3^ (geometric mean) before vision testing, and 700 μg/m^3^ (geometric mean) at the time of vision testing (NYSDOH unpublished data). Based on these reports, effects on cognitive and/or visual function might be hypothesized to occur among individuals exposed to the levels of perc encountered in some apartments included in this study. Visual function assessments (VCS, color vision) and biologic measures of exposure (blood, breath perc levels) have been obtained for participants in the NYC Perc Project. Analyses of these data will allow us to relate environmental and biologic measures of perc exposure to each other and to the occurrence of visual function effects. This, in turn, will allow us to assess whether the evident inequity in perc exposure documented here contributes to an inequity in health outcome.

## Conclusions

Mean indoor air perc levels in residential dry cleaner buildings in New York City (Manhattan) have decreased by about 10-fold since 1997, when additional dry cleaner regulations were adopted to reduce and contain fugitive perc emissions. By 2001–2003, the mean apartment perc level was 34 μg/m^3^, 10-fold lower than mean apartment levels of 340–360 μg/m^3^ documented before 1997. The maximum detected perc level was 5,000 μg/m^3^, 5-fold lower than the maximum of 25,000 μg/m^3^ documented before 1997. Despite these accomplishments, many residences in dry cleaner buildings still have levels above the NYSDOH air guideline of 100 μg/m^3^, and some have levels above 1,000 μg/m^3^. Moreover, buildings located in low-income and minority neighborhoods have disproportionately elevated perc levels. Logistic regression suitable for clustered data indicated that apartment indoor air perc level is significantly more likely to exceed 100 μg/m^3^ in dry cleaner buildings located in minority neighborhoods (OR = 6.7; 95% CI, 1.5–30.5) compared with buildings located in nonminority neighborhoods. Factors that may be contributing to the elevated perc levels detected, especially in minority and low-income neighborhoods, are being explored.

## Figures and Tables

**Table 1:** Summary of buildings and households sampled, by predominant population.

	Buildings with on-site dry cleaners			Buildings without dry cleaners		
	Minority low income	Nonminority higher income	Total	Minority low income	Nonminority higher income	Total
Buildings						
Identified^a^	16	164	180	—	—	—
Characterized^b^	16	120	136	—	—	—
Met criteria^c^	11	72	83	57	236	293
Contacted^d^	11	56	67	—	—	—
Sampled^e^	8	16 (9)^f^	24	15	21	36
Apartments						
Identified^g^	169	2,611	2,780	485	2,730	3,215
Contacted^h^	102	1,159	1,261	273	979	1,252
Potentially eligible^i^	31	101	132	63	112	175
Eligible j	23	66	89	29	51	80
Participated^k^	22	43 (21)^l^	65	22	39	61

—, not applicable.

a Dry cleaners reporting using perc on site.

b Dry cleaner buildings surveyed for presence of occupied residences; absence of other VOC sources.

c Dry cleaner buildings with occupied residences; no other VOC sources.

d Dry cleaner building where household recruitment was attempted.

e Dry cleaner building where at least one apartment was sampled.

f Number of buildings that had received a prior resident complaint.

g Estimated total apartments present.

h Presence of age-eligible child(ren) determined.

i Age-eligible adult and child present.

j Met screening level NYC Perc Project household inclusion criteria.

k Apartment indoor air sampled for perc.

l Number of apartments located in buildings that had received a prior complaint.

**Table 2:** Perc levels (μg/m3) in residential dry cleaner buildings.

	Building census block group category					Perc (μg/m3)	
Building designation	Low income	Minority	Building prior complaint	No. of floors	Floor(s) sampled	Mean apartment level^a^	Maximum building level
e368			X	15	14	5 (PL)	5 (PL)
e702		X		6	1, 4, 5, 6	5 (PL), 5 (PL), 5 (PL), 10	10
e56				14	3, 3	5 (PL), 12	12
e103				11	7	13	13
e369			X	4	3	27	27
e107			X	11	5, 11, 11, 11	8, 28, 13, 39	39
e41		X		16	15, 16, 16	9, 42, 10	42
e432			X	17	15, 15	49, 36	49
e53				26	3, 5	61, 8	61
e63				16	4, 5, 7, 10, 17, 17	5 (PL), 5 (PL), 5 (PL), 5 (PL), 80, 13	80
e252			X	6	1	84	84
e64		X		13	3, 6, 7, 8	99, 5 (PL), 28, 22	99
e47			X	12	2, 3, 4, 5, 6, 8, 11	5 (PL), 12, 92, 5 (PL), 25, 69, 194	194
e703	X	X		7	1, 3, 4, 6, 7, 7	216, 41, 130, 12, 45, 78	216
e404			X	16	2, 2, 3	5 (PL), 322, 5 (PL)	322
e249			X	4	2	352	352
e431			X	7	2	372	372
e152				13	2, 7, 8, 11	400, 5 (PL), 15, 17	400
e18a		X		4	3	695	695
e4	X	X		4	3	760	760
e6	X	X		4	2, 4	215, 2,100	2,100
e700	X	X		3	3	2,135	2,135
e22		X		6	1, 4, 4, 4, 4, 6	84, 710, 4,600, 225, 335, 8	4,600
e5	X	X		4	3	5,000	5,000

a Mean of duplicate values for main living space; quantities of perc PL were assigned half the detection limit (2.5 μg/m3) for all quantitative analysis. Perc values correspond to floors sampled.

**Table 3:** Summary of apartments and buildings sampled.

	No.	Percent
Apartments sampled	65	
Mean < background (11 μg/m3)	21	32
Background (11 μg/m3) < mean ≤100 μg/m3	27	42
100 μg/m3 < mean ≤1,000 μg/m3	13	20
Mean > 1,000 μg/m3	4	6
Buildings sampled	24	
Building maximum < background (11 μg/m3)	2	8
Background (11 μg/m3) < building maximum ≤100 μg/m3	10	42
100 μg/m3 < building maximum ≤1,000 μg/m3	8	33
Building maximum > 1,000 μg/m3	4	17

**Table 4:** Current and previously reported perc levels (μg/m3) in apartments and buildings with and without dry cleaners.

						Perc (μg/m3)^a^		
Study (location)	Sampling period	Dry cleaner type	Buildings sampled	Apartments sampled	GM	Median	Range
Current NYC Perc Project (New York City)	2001–2003	On-site	24	65	35	28	3–5,000
		Minority	10	29	75	78	3–5,000
		Nonminority	14	36	19	14	3–400
		Low income	5	11	256	215	12–5,000
		Higher income	19	54	23	16	3–4,600
		Drop-off	5	9	6	3	3–29
		None	36	61	3	3	3–92
Before adoption of state dry cleaner regulations (NYSDEC 1997)							
NYSDOH, unpublished data (New York City)^b^	1996–1997	On site	8	18	336	530	19–5,500
Wallace et al. 1995 (New York City)	1994–1995	On site	12	29	361	441	7–25,000
		None	8	10	3	6	1–19
NYSDOH, unpublished data (New York City)	1991–1993	On-site, morning	16	20	1,326	2,091	6–24,667
		On-site, evening	1	5	4,629	5,900	400–48,000
Schreiber et al. 1993 (Albany, NY)	1991–1992	On-site, morning	6	6	3,061	2,790	300–55,000
		On-site, evening	6	6	212	4,865	100–36,500
		None, morning	6	6	35	44	10–103
		None, evening	6	6	46	56	22–77

GM, geometric mean.

a Values below the detection limit (5 μg/m3) were assigned one-half the detection limit (2.5 μg/m3) before log transformation and derivation of summary statistics; sampling times varied by study and ranged from 4 to 24 hr.

b Subset of buildings included in Schreiber et al. (2002).
